# Genetic and epigenetic associations of *ANRIL* with coronary artery disease and risk factors

**DOI:** 10.1186/s12920-021-01094-8

**Published:** 2021-10-06

**Authors:** Bayi Xu, Zhixia Xu, Yequn Chen, Nan Lu, Zhouwu Shu, Xuerui Tan

**Affiliations:** 1 Department of Cardiology, First Affiliated Hospital of Shantou University Medical College, Shantou, 515041 Guangdong China; 2 Department of Medical Service, Second Affiliated Hospital of Shantou University Medical College, Shantou, 515041 Guangdong China

**Keywords:** *ANRIL*, Coronary artery disease, Single nucleotide polymorphisms, DNA methylation

## Abstract

**Background:**

Both DNA genotype and methylation of antisense non-coding RNA in the INK4 locus (*ANRIL*) have been robustly associated with coronary artery disease (CAD), but the interdependent mechanisms of genotype and methylation remain unclear.

**Methods:**

Eighteen tag single nucleotide polymorphisms (SNPs) of *ANRIL* were genotyped in a matched case–control study (cases 503 and controls 503). DNA methylation of *ANRIL* and the INK4/ARF locus (*p14*^*ARF*^, *p15*^*INK4b*^ and *p16*^*INK4a*^) was measured using pyrosequencing in the same set of samples (cases 100 and controls 100).

**Results:**

Polymorphisms of *ANRIL* (rs1004638, rs1333048 and rs1333050) were significantly associated with CAD (*p* < 0.05). The incidence of CAD, multi-vessel disease, and modified Gensini scores demonstrated a strong, direct association with *ANRIL* gene dosage (*p* < 0.05). There was no significant association between *ANRIL* polymorphisms and myocardial infarction/acute coronary syndrome (MI/ACS) (*p* > 0.05). Methylation levels of *ANRIL* were similar between the two studied groups (*p* > 0.05), but were different in the rs1004638 genotype, with AA and AT genotype having a higher level of *ANRIL* methylation (pos4, *p* = 0.006; pos8, *p* = 0.019). Further Spearman analyses indicated that methylation levels of *ANRIL* were positively associated with systolic blood pressure (pos6, *r* = 0.248, *p* = 0.013), diastolic blood pressure (pos3, *r* = 0.213, *p* = 0.034; pos6, *r* = 0.220, *p* = 0.028), and triglyceride (pos4, *r* = 0.253, *p* = 0.013), and negatively associated with high-density lipoprotein cholesterol (pos2, *r* = − 0.243, *p* = 0.017). Additionally, we identified 12 transcription factor binding sites (TFBS) within the methylated *ANRIL* region, and functional annotation indicated these TFBS were associated with basal transcription. Methylation at the INK4/ARF locus was not associated with *ANRIL* genotype.

**Conclusions:**

These results indicate that *ANRIL* genotype (tag SNPs rs1004638, rs1333048 and rs1333050) mainly affects coronary atherosclerosis, but not MI/ACS. There may be allele-related DNA methylation and allele-related binding of transcription factors within the *ANRIL* promoter.

**Supplementary Information:**

The online version contains supplementary material available at 10.1186/s12920-021-01094-8.

## Background

Current genome-wide association studies (GWAS) have added a considerable number of loci to serve as genetic markers of coronary artery disease (CAD) and myocardial infarction (MI)/acute coronary syndrome (ACS). Loci most frequently replicated in independently unbiased GWAS are at Chr9p21.3, Chr6p24.1, and Chr1p13.3. Chr9p21.3 stands out for its relatively large effect size, high allele frequency of more than 50% [1, 2] and ethnic diversity [3–5]. However, the causative gene for CAD at this locus is unknown. The CAD core risk region on 9p21.3 harbors no coding genes, but expresses the long non-coding RNA antisense non-coding RNA in the INK4 locus (*ANRIL*). The closest adjacent protein-coding genes are in the INK4/ARF locus, which encodes the key tumor suppressors, *p14*^*ARF*^, *p15*^*INK4b*^ and *p16*^*INK4a*^, and are considered as potential functional candidates [6].

Most of the 9p21.3 CAD-associated genomic variants are located within *ANRIL* [7], also, expression of *ANRIL* has been shown to associated with CAD and MI [8, 9], which makes *ANRIL* the most robust genetic marker of CAD today. However, inconsistent results have been reported [10]. Whether *ANRIL* polymorphisms are associated with the likelihood of CAD and its main effect (coronary atherosclerosis or plaque instability) on CAD remains controversial [11]. As an effector gene in 9p21.3, *ANRIL* can regulate its adjacent INK4/ARF locus *in cis,* as well as the distant loci *in trans* [9, 10, 12]. Causal variants at *ANRIL* can disrupt predicted transcription factor binding sites (TFBS) [13] and modulate *ANRIL* expression and/or structure [9, 14, 15], playing a pivotal role in mediating the 9p21.3 susceptibility for CVD. On the other hand, epigenetic modification, such as DNA methylation, at the *ANRIL* and INK4/ARF loci has been implicated in the genetic cause of CVD [16–19]. In cancer cells, transcription of *ANRIL* and *p16*^*INK4a*^ is regulated by the methylation status of *p16*^*INK4a*^ [20]. However, the relationship between polymorphism of *ANRIL* and DNA methylation of *ANRIL* and the INK4/ARF locus in CAD patients has not been examined. In this study, we systematically examined the polymorphisms of *ANRIL* using haplotype tag SNPs and detected their associations with CAD risk and the severity of coronary atherosclerosis, and then explored the potential relationship between the polymorphism of *ANRIL* and DNA methylation of *ANRIL* and the INK4/ARF locus in a Chinese population.

## Methods

### Subjects

Angiographic CAD was determined by blinded coronary angiographic analysis and defined as having > 50% diameter stenosis in at least 1 major epicardial coronary artery. Modified Gensini coronary scores were used to assess the severity of CAD [21, 22]. For a vessel to be scored, stenosis > 50% had to be noted in an epicardial coronary vessel of its major branches [23].

Subjects with other cardiac diseases (congenital heart disease, cardiomyopathy, or rheumatic heart disease), cerebrovascular or neurological diseases, cancer, severe liver or kidney disease were excluded from the study. Furthermore, patients who had received angioplasty, intravenous thrombolysis, coronary artery stents, or coronary artery bypass graft surgery before the enrollment were also excluded.

Finally, 503 angiographic CAD cases and 503 age- (3-year bands) and sex-matched controls were selected from consecutive patients undergoing diagnostic or interventional coronary angiography within the First Affiliated Hospital of Shantou University Medical College from April 20, 2015 to August 20, 2016. One hundred eighty-eight MI/ACS and 188 age- (3-year bands) and sex-matched angiographic CAD controls (CAD patients without MI/ACS) were selected according to the third universal definition of myocardial infarction [24] from the same hospital during the same period. The controls were free of MI/ACS by questionnaires, history-taking, detection of troponin-T and myocardial enzymes, electrocardiography, chest X-ray, and Doppler echocardiography.

Demographic and clinical data required for this study were obtained from physician and hospital records, and included age, sex, health history (CAD, MI/ACS, hypertension, type 2 diabetes, hyperlipidemia), vital signs at entry, medication use, personal hobbies (smoking, alcohol use), total cholesterol (TC), low-density lipoprotein cholesterol (LDL), high-density lipoprotein cholesterol (HDL), triglycerides (TG), glycosylated hemoglobin (HbAlc), creatinine, uric acid, and systolic and diastolic blood pressure (SBP and DBP).

All subjects were from the Chinese Han population and gave informed consent prior to the study. Ethics approval was obtained from the Ethics Committee of the First Affiliated Hospital of Shantou University Medical College.

### SNP selection and genotyping

An arterial blood sample was taken at the time of the catheterization for deoxyribonucleic acid extraction and subsequent genotyping. Genomic deoxyribonucleic acid was isolated with a FlexGen Blood DNA Kit according to the manufacturer’s protocol (CoWin Biosciences). Eighteen tag SNPs of *ANRIL* were selected by Haploview software (Version 4.1). The minor allele frequency of each SNP was > 5% in the HapMap of the Chinese Han Beijing (CHB) population (see Additional file 1: Table S1).

Genotyping was performed with the Access Array micro-fluidics PCR platform (Fluidigm Corporation, South San Francisco, California, USA) according to the standard instructions [25]. Fluidigm SNP Genotyping Analysis (Version 4.3.2) software was used for data management and analyses.

### Methylation analysis of *ANRIL*, *p14*^*ARF*^, *p15*^*INK4b*^ and *p16*^*INK4a*^

Genomic DNA was isolated as mentioned above. Bisulfite treatment of DNA was done by using the EZ DNA MethylationTM kit (ZYMO Research, Orange, CA) according to the manufacturer’s protocol. Then the resulting bisulfite-treated DNA was purified and eluted in 20 μl of M-elution buffer, and 4 μl of this was used in the methylation-specific PCR (MSP) amplification. Primers for MSP amplification were designed with the use of the bioinformatics program (http://www.urogene.org/methprimer/index1.html) and are shown in Additional file 1: Table S2. After amplification, the MSP products were analyzed by bisulfite pyrosequencing (PyroMark Q96 System version 2.0.6, Qiagen).

### Functional annotation of the *ANRIL* methylation region

PROMO (version 8.3 of TRANSFAC) was used to predict the putative TFBS within the *ANRIL* methylation region. The Database for Annotation, Visualization and Integrated Discovery (DAVID) v6.8 was used to annotate the function of the predicted TFBS. Pathways that were potentially affected by gene DNA methylation were generated by Kyoto Encyclopedia of Genes and Genomes (KEGG) enrichment analysis.

### Statistical analysis

SPSS 19.0 (IBM Corp© 2010) and SNPassoc for R statistical package were used for statistical analyses. Haploview software (version 4.1) was used for analyses of the pairwise linkage disequilibrium, haplotype structure and selecting tagging SNPs. All *p-*values were two sided, and *p* < 0.05 was considered statistically significant.

Continuous variables were reported as the mean value ± standard deviation (SD). Data with a normal distribution were compared by Student *t* test or ANOVA test, and those with unequal variance or without a normal distribution were analyzed by a Mann–Whitney rank sum test or Spearman correlation test. Categorical variables were expressed as frequencies with percentages and were compared by the chi-squared (*χ*^2^) test. A trend test was also examined by the *χ*^*2*^ test (linear-by-linear association). Genotypic frequencies in cases and controls were tested for departure from Hardy–Weinberg equilibrium using a Fisher’s exact test. Genetic model analyses (dominant, recessive) were applied to assess the significance of SNPs, and the allelic frequencies were compared between cases and controls by *χ*^2^/Fisher’s exact test. The associations between CAD and genotypes of the SNPs were estimated by computing the odds ratios (ORs) and 95% confidence intervals (CIs) from the multivariable logistic regression, and adjusted for sex, age, smoking, alcohol use, hypertension, type 2 diabetes, blood lipid (TC, TG, HDL, LDL), creatinine and uric acid.

## Results

### General characteristics of the subjects

The main demographic and clinical characteristics of the CAD cases and controls are summarized in Table 1. Mean age of the cases was 59.67 years old (from 39 to 87), mean age of the controls was 60.42 years old (from 33 to 85), there was no significant difference between the two groups (*p* = 0.198). As expected, other traditional risk factors, such as hypertension, type 2 diabetes and hyperlipidemia were more prevalent in cases than the controls (*p* < 0.05). Accordingly, mean levels of SBP, HbAlc, TG and HDL were significantly different between the two groups (*p* < 0.05). DBP, TC and LDL did not differ between the cases and controls (*p* > 0.05), which could be the result of antihypertensive and cholesterol-lowering drugs in the patients after diagnosis.

**Table 1:** General characteristics of the CAD cases and controls

Variables	Case (n = 503)	Control (n = 503)	*P*-value
Categorical variables, n (%)			
Male	311 (61.83)	291 (57.85)	0.222
Smoking	212 (42.15)	180 (35.79)	0.047
Alcohol use	52 (10.34)	44 (8.75)	0.396
Hypertension	390 (77.53)	317 (63.02)	< 0.001
Type 2 diabetes	258 (51.29)	149 (29.62)	< 0.001
Hyperlipidemia	347 (68.99)	303 (60.24)	0.006
Continuous variables, mean ± SD			
Age (years)	59.67 ± 9.85	60.42 ± 9.08	0.198
SBP (mmHg)	139.47 ± 23.09	136.57 ± 20.91	0.037
DBP (mmHg)	82.93 ± 12.82	83.38 ± 12.80	0.583
HbAlc (%)	5.66 ± 2.92	4.66 ± 2.82	< 0.001
TC (mmol/L)	4.76 ± 1.38	4.87 ± 1.09	0.383
TG (mmol/L)	1.60 ± 1.33	1.41 ± 0.90	< 0.001
HDL (mmol/L)	1.09 ± 0.32	1.20 ± 0.30	< 0.001
LDL (mmol/L)	3.16 ± 1.04	3.24 ± 0.85	0.594
Creatinine (μmol/L)	104.08 ± 86.98	94.15 ± 31.89	0.017
Uric acid (μmol/L)	397.46 ± 109.56	383.26 ± 111.30	0.048

CAD: coronary artery disease; SD: standard deviation; SBP: systolic blood pressure; DBP: diastolic blood pressure; HbA1c: hemoglobin A1c; TC: total cholesterol; TG: triglyceride; HDL: high-density lipoprotein cholesterol; LDL: low-density lipoprotein cholesterol

### Association of *ANRIL* genotype with CAD

Of the 18 tag SNPs, rs10965227 and rs10965241 did not conform to the Hardy–Weinberg equilibrium test in controls and cases (see Additional file 1: Table S1), and so were not used for further analysis. Univariate analyses found 5 SNPs (rs1004638, rs1333048, rs1333050, rs4977756, rs9632885) to be significantly associated with CAD (see Additional file 1: Table S3), while the other 11 SNPs had no association with CAD (data not shown).

In multivariable logistic regression analysis, after adjusting for conventional CAD risk factors such as sex, age, smoking, alcohol use, hypertension, type 2 diabetes and hyperlipidemia, we found 3 SNPs (rs1004638, rs1333048 and rs1333050) significantly associated with CAD (Table 2). rs1004638 showed a large effect size both in heterozygotes (AT, OR = 2.13, 95% CI 1.34–3.40) and homozygotes (AA, OR = 2.50 (95% CI 1.58–3.95). rs1333048 showed a modest effect size in heterozygotes (AC, OR = 1.38, 95% CI 1.01–1.88) and was exaggerated in homozygotes (CC, OR = 2.02 (95% CI 1.40–2.91). rs1333050 showed no effect size in heterozygotes (CT, OR = 1.21, 95% CI 0.88–1.66), but was exaggerated in homozygotes (TT, OR = 1.58 (95% CI 1.09–2.28). The combined rs1004638 AA + AT genotypes (OR = 2.32, 95% CI 1.49–3.62), rs1333048 AC + CC genotypes (OR = 1.56, 95% CI 1.16–2.09), and rs1333050 CT + TT genotypes (OR = 1.32, 95% CI 0.97–1.78) had 2.32-, 1.56-, and 1.32-fold higher CAD risk respectively, compared with the rs1004638 TT, rs1333048 AA, and rs1333050 CC genotypes. For the risk allele A, C, T of the three SNPs, the adjusted ORs of CAD were 1.39 (95% CI 1.15–1.69), 1.40 (95% CI 1.17–1.67), and 1.24 (95% CI 1.04–1.48), respectively. There were dose–response effects of rs1004638 A, rs1333048 C, and rs1333050 T alleles with CAD (*P*_trend_ = 0.008, 0.001, and 0.027, respectively).

**Table 2:** Odds ratios of *ANRIL* tag SNPs for CAD by multivariable logistic regression analysis

SNP	Model		Cases (n)	Controls (n)	Adjusted OR (95% CI)	*P* value
rs1004638	Genotype	TT (Ref.)	27	68	1	
		AT	206	208	2.13 (1.34–3.40)	0.001
		AA	268	225	2.50 (1.58–3.95)	< 0.001
		AT + AA	474	433	2.32 (1.49–3.62)	< 0.001
	*P*_trend_					0.008
	Allele	T (Ref.)	260	344	1	
		A	742	658	1.39 (1.15–1.69)	< 0.001
rs1333048	Genotype	AA (Ref.)	104	141	1	
		AC	249	259	1.38 (1.01–1.88)	0.044
		CC	147	97	2.02 (1.40–2.91)	< 0.001
		AC + CC	396	356	1.56 (1.16–2.09)	0.003
	*P*_trend_					0.001
	Allele	A (Ref.)	457	541	1	
		C	543	453	1.40 (1.17–1.67)	< 0.001
rs1333050	Genotype	CC (Ref.)	101	124	1	
		CT	259	265	1.21 (0.88–1.66)	0.242
		TT	142	109	1.58 (1.09–2.28)	0.015
		CT + TT	401	374	1.32 (0.97–1.78)	0.074
	*P*_trend_					0.027
	Allele	C (Ref.)	461	513	1	
		T	541	483	1.24 (1.04–1.48)	0.018

Ref.: reference variable; CAD: coronary artery disease; OR: odds ratio; CI: confidence interval. Trend test was examined by χ^2^ test (linear-by-linear association)

The linkage disequilibrium was defined as D ≥ 95, and five haplotype blocks were identified in the *ANRIL* region (see Additional file 1: Fig. S1). Haplotype analysis found blocks 1, 2, 3, and 5 were not significantly associated with CAD. rs1004638 and rs1333048 showed strong linkage in block 4, and the haplotype AC (A, C are the risk alleles of rs1004638 and rs1333048, respectively) was significantly associated with an increased risk of CAD (OR = 1.42, 95% CI 1.22–1.66; *p* < 0.001).

### Effect of *ANRIL* genotype on CAD severity and MI/ACS

There was a strong positive association between the incidence of CAD and increasing gene dose of the rs1004638 (*p* < 0.001), rs1333048 (*p* < 0.001) and rs1333050 (*p* = 0.034) risk variants. Multi-vessel disease (*p* = 0.027) increased as increasing gene dosage of rs1333048 risk variant. Gensini scores in the mutant homozygote of rs1333048 (CC, *p* < 0.001) and rs1333050 (TT, *p* = 0.001) were significantly higher than that of the heterozygote or wild-type homozygote. Patients with two hazardous alleles of *ANRIL* were more likely to suffer severe CAD (Table 3).

**Table 3:** Association of gene dosage of *ANRIL* tag SNPs with CAD severity

SNP	Genotype/*p*-value	CAD, n (%)		Criminal Vessels, n (%)		Gensini scores	
		Yes	No	Multi-VD	1-VD	n	Mean ± SD
rs1004638	TT	27 (28.42)	68 (71.58)	15 (55.56)	12 (44.44)	27	37.17 ± 34.28
	AT	206 (49.76)	208 (50.24)	131 (63.59)	75 (36.41)	205	37.36 ± 32.62
	AA	268 (54.36)	225 (45.64)	183 (68.28)	85 (31.72)	268	41.93 ± 34.08
	*P*	< 0.001		0.296		0.313	
rs1333048	AA	104 (42.45)	141 (57.55)	63 (60.58)	41 (39.42)	104	34.47 ± 30.52
	AC	249 (49.02)	259 (50.98)	158 (63.45)	91 (36.55)	248	36.69 ± 32.78
	CC	147 (60.25)	97 (39.75)	107 (72.79)	40 (27.21)	147	49.01 ± 35.16
	*P*	< 0.001		0.027		< 0.001	
rs1333050	CC	101 (44.89)	124 (55.11)	66 (65.35)	35 (34.65)	101	36.96 ± 32.39
	CT	259 (49.43)	265 (50.57)	162 (62.55)	97 (37.45)	258	36.11 ± 31.51
	TT	142 (56.57)	109 (43.43)	102 (71.83)	40 (28.17)	142	48.61 ± 36.24
	*P*	0.034		0.172		0.001	

CAD: coronary artery disease; Multi-VD: multi-vessel disease; 1-VD: 1-vessel disease. SD: standard deviation

In addition, we performed a stratified analysis by age. Premature CAD (PCAD) in this study was defined as CAD occurring in males < 55 years of age and females < 65 years of age [26]. Accordingly, late-onset CAD (LCAD) was defined as males ≥ 55 years of age and females ≥ 65 years of age. Multivariable logistic regression analysis showed that there were appreciably higher ORs for the risk alleles in the PCAD set (*p* < 0.05) when compared with the LCAD set (*p* ≥ 0.05) (Table 4).

**Table 4:** Odds ratios of *ANRIL* tag SNPs for PCAD and LCAD by multivariable logistic regression analysis

SNP	Genotype	PCAD		LCAD	
		Adjusted OR (95% CI)	*p* value	Adjusted OR (95% CI)	*p* value
rs1004638	TT (Ref.)	1.00		1.00	
	AT	6.32 (2.27–17.61)	< 0.001	1.98 (1.01–3.87)	0.047
	AA	6.14 (2.19–17.16)	0.001	1.43 (0.73–2.83)	0.301
rs1333048	AA (Ref.)	1.00		1.00	
	AC	2.19 (1.26–3.78)	0.005	0.88 (0.56–1.38)	0.568
	CC	2.77 (1.46–5.24)	0.002	1.55 (0.92–2.60)	0.098
rs1333050	CC (Ref.)	1.00		1.00	
	CT	1.65 (0.96–2.85)	0.072	1.03 (0.69–1.55)	0.873
	TT	2.30 (1.21–4.36)	0.011	1.28 (0.81–2.02)	0.296

Ref.: reference variable; PCAD: premature CAD; LCAD: late-onset CAD; OR: odds ratio; CI: confidence interval

To explore the effect of *ANRIL* on plaque instability, another case–control study (188 MI/ACS and 188 age- (3-year bands) and sex-matched CAD controls) was carried out. The results showed that there was no association between *ANRIL* tag SNPs and MI/ACS (*p* > 0.05) (see Additional file 1: Table S4).

### Methylation of *ANRIL*, *p14*^*ARF*^, *p15*^*INK4b*^ and *p16*^*INK4a*^ in the study population

**Fig. 1:**
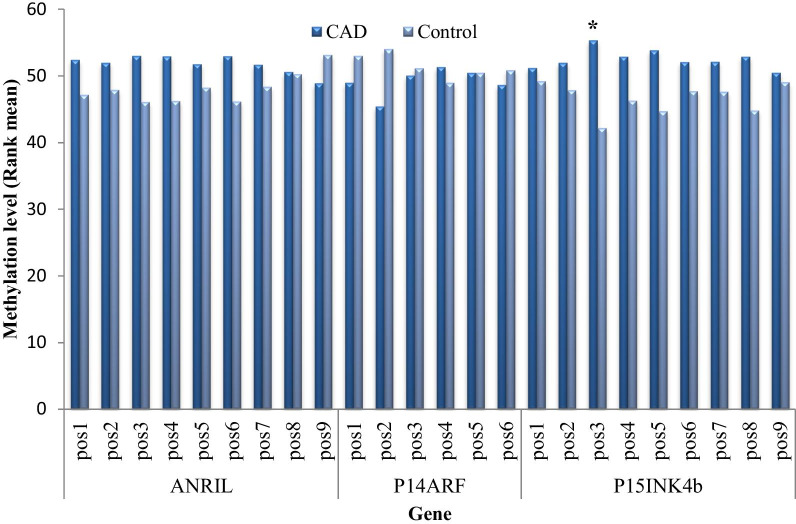
Comparison of methylation level of *ANRIL*, *P14*^*ARF*^ and *P15*^*INK4b*^ in CAD patients and controls. pos: methylation sites; *p value < 0.05

To further explore the possible molecular mechanism of CAD, we assessed DNA methylation of the *ANRIL, p14*^*ARF*^*, p15*^*INK4b*^ and *p16*^*INK4a*^ promoter region in CAD and control subjects. The one-sample K–S test was used to test the normality of DNA methylation levels, and it was found that the data did not conform to a normal distribution, so the Mann Whitney rank sum test was used to compare the differences between groups. As shown in Fig. 1, only one CpG site within the third CpG island (pos3) located upstream of *p15*^*INK4b*^ was hyper-methylated in CAD subjects compared to the matched controls (*p* = 0.025). No differences in *ANRIL* or *p14*^*ARF*^ methylation were observed (*P* > 0.05). Methylation of *p16*^*INK4a*^ was at barely detectable levels in both CAD patients and controls, so the data was not shown.

### Association of *ANRIL* genotype with methylation

**Fig. 2:**
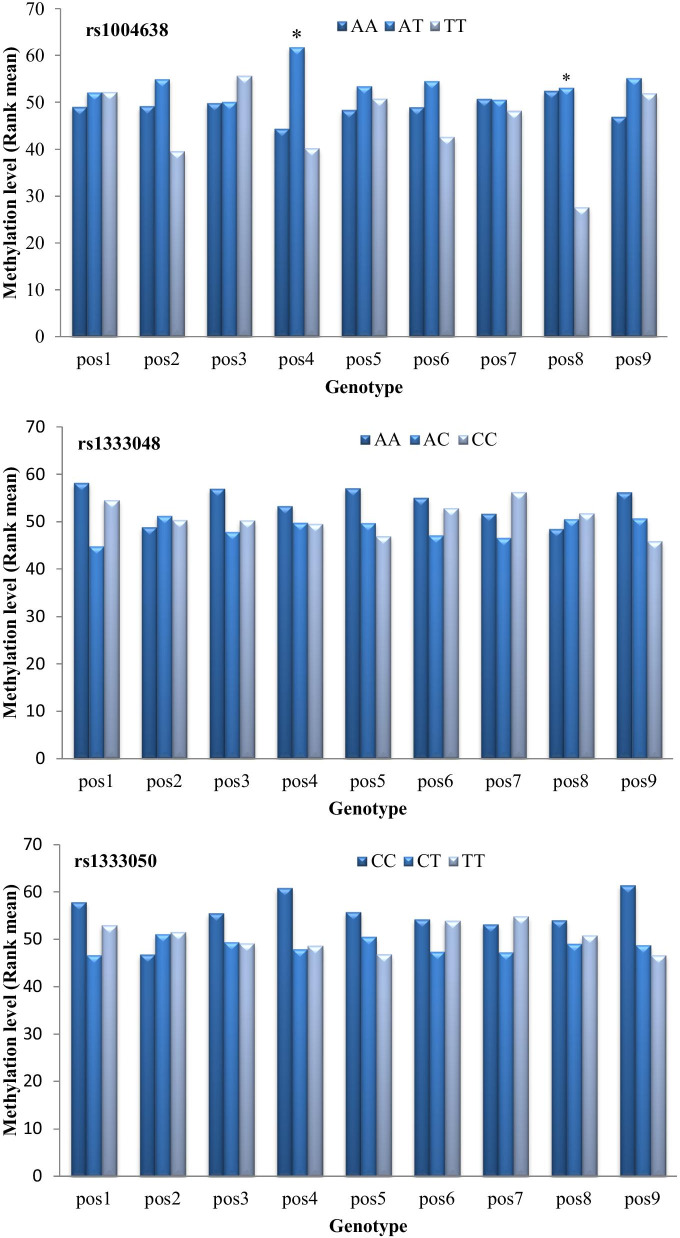
Association of *ANRIL* polymorphisms (rs1004638, rs1333048, rs1333050) with *ANRIL* methylation. pos: methylation sites; *p value < 0.05

Further, we assessed the association between CAD risk genotypes and methylation of *ANRIL, p14*^*ARF*^ and *p15*^*INK4b*^. CAD-associated rs1004638 was associated with methylation of *ANRIL*. Compared with carriers of TT genotypes, AA and TT genotype carriers of rs1004638 had markedly elevated levels of methylation [*ANRIL* pos4 (*p* = 0.006) and pos8 (*p* = 0.019)]. rs1333048 and rs1333050 had no effect on the methylation level of *ANRIL* (Fig. 2). There were no differences between CAD-associated SNPs (rs1004638, rs1333048, rs1333050) and methylation of *p14*^*ARF*^ or *p15*^*INK4b*^ (data not shown).

### Correlation between *ANRIL* methylation and risk factors of CAD

**Fig. 3:**
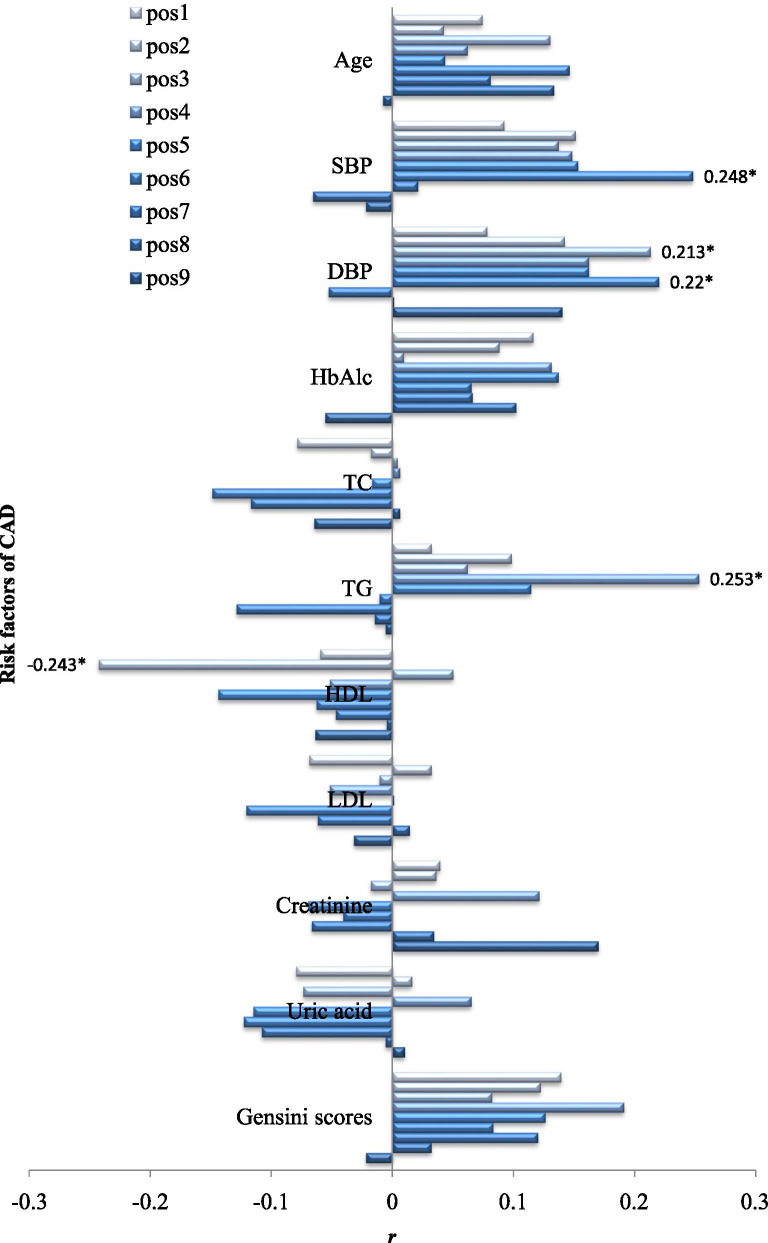
Correlation between *ANRIL* methylation and risk factors for CAD. Correlation coefficients with statistical significance are listed in the figure. pos: methylation sites; *p value < 0.05; *r*: Spearman correlation coefficient; SBP: systolic blood pressure; DBP: diastolic blood pressure; HbA1c: hemoglobin A1c; TC: total cholesterol; TG: triglyceride; HDL: high-density lipoprotein cholesterol; LDL: low-density lipoprotein cholesterol

Figure 3 shows the results of correlation analyses between *ANRIL* methylation and risk factors of CAD. Spearman analyses indicated that *ANRIL* methylation levels were positively associated with SBP (pos6, *r* = 0.248, *p* = 0.013), DBP (pos3, *r* = 0.213, *p* = 0.034; pos6, *r* = 0.220, *p* = 0.028), and TG (pos4, *r* = 0.253, *p* = 0.013), and negatively associated with HDL (pos2, r = − 0.243, *p* = 0.017).

### Functional annotation of *ANRIL* methylation region

**Fig. 4:**
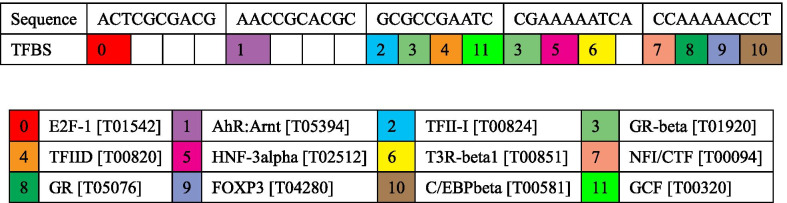
Predicted TFBS in DNA sequences within the *ANRIL* methylated region. Numbers in the colored boxes of the upper panel refer to the binding site of different transcription factors. E2F: transcription factor 1 (Gene ID: 1869); AhR: aryl hydrocarbon receptor (Gene ID: 196); Arnt: aryl hydrocarbon receptor nuclear translocator (Gene ID: 405); TFII-I: general transcription factor Iii (Gene ID: 2969); GR-beta: nuclear receptor subfamily 3 group C member 1 (Gene ID: 2908); TFIID: TATA box-binding protein (Gene ID: 6908); HNF-3alpha: forkhead box A1 (Gene ID: 3169); T3R-beta1: thyroid hormone receptor alpha (Gene ID: 7067); NFI/CTF: nuclear factor I C (Gene ID: 4782); GR: nuclear receptor subfamily 3 group C member 1 (Gene ID: 2908); FOXP3: forkhead box P3 (Gene ID: 50943); C/EBPbeta: CCAAT enhancer binding protein beta (Gene ID: 1051); GCF: GC-rich sequence DNA-binding factor 2 (Gene ID: 6936)

**Fig. 5:**
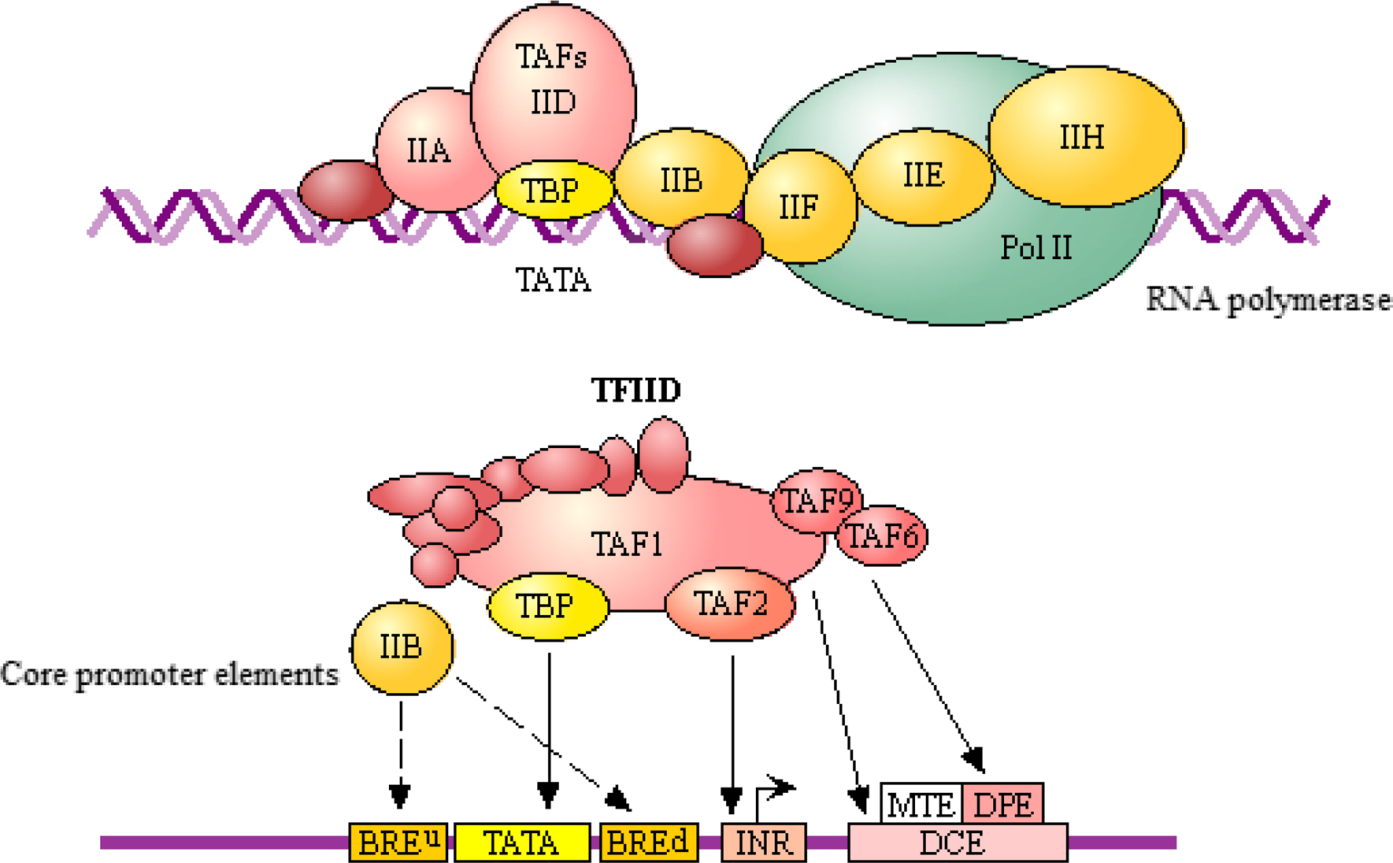
KEGG pathway enrichment analysis of the *ANRIL* methylated region. General transcription factors for RNA polymerase II. TFIID is composed of TATA-binding protein (TBP) and a number of TBP-associated factors (TAFs). Pol II: RNA polymerase II; TAF: TATA box-binding protein-associated factor; TBP: TATA box-binding protein; BRE: TFIIB recognition element; DPE: downstream promoter element; INR: initiator; DCE: downstream core components; MTE: motif ten elements

We predicted 12 TFBS in DNA sequences within the *ANRIL* methylation region (Fig. 4). Functional annotation by KEGG pathway enrichment analysis indicated these TFBS-binding sites were associated with basal transcription (Fig. 5). Among them, transcription factor IID (TFIID) was identified as a key transcription factor, which is composed of the TATA-binding protein and binds to the core promoter to position the RNA polymerase II properly, serves as the scaffold for assembly of the remainder of the transcription complex, and acts as a channel for regulatory signals [27–30].

## Discussion

In the present study, we demonstrated that: (1) tag SNPs (rs1004638, rs1333048, rs1333050) of *ANRIL* significantly influence the hazard of CAD in a Chinese Han population. Homozygous carriers showed higher coronary atherosclerosis risk, whereas heterozygous carriers showed intermediate risk between that of wild-type and homozygous carriers, indicating a genetic dose effect, especially for premature CAD. (2) *ANRIL* polymorphism was significantly associated with CAD severity, but not MI/ACS. (3) DNA methylation levels of *ANRIL* were not associated with CAD, but were associated with rs1004638 and CAD risk factors (SBP, DBP, TG and HDL). (4) Twelve TFBS were predicted within the *ANRIL* methylation region. These findings indicate that there may be allele-related DNA methylation and allele-related binding of transcription factors within the *ANRIL* promoter region.

Our results are consistent with previous studies conducted in China and other populations in which *ANRIL* polymorphisms were found to be associated with CAD [11, 31]. A quantitative assessment of the severity of coronary stenosis is better than a binary phenotype [32], while previous studies found that *ANRIL* confers risk for CAD vs. controls. So, a validated semi-quantitative angiographic score, Gensini scores, was used to estimate severity of CAD in this study. Our results showed a dose–effect relationship between the Gensini scores and *ANRIL* risk alleles. Homozygotes of the rs1333048 risk allele are more likely (72.79%) to suffer multi-vessel disease than wild-type carriers (60.58%), thus providing clues for predicting severity of CAD.

Considering that *ANRIL* is related to the degree of coronary stenosis, we wondered whether it is also related to MI/ACS. MI/ACS patients are prone to suffer multiple vessel lesions [23]. Over 50% of ST-segment elevation MI patients have multiple vessel lesions, which indicates poor prognosis [33]. However, our results demonstrate that there is no association between *ANRIL* and MI/ACS when both cases and controls have coronary stenosis. Such an analytical method has been used in previous studies as a means to distinguish the effect of genetic factors on CAD from MI [34]. However, by comparing MI cases with healthy controls, Abdul Azeez et al*.* [35] and Cheng et al*.* [36] did not distinguish the effect of *ANRIL* on CAD and MI, but showed that *ANRIL* is associated with MI in both the Saudi population and Chinese Han population. Given that MI is a more downstream phenotype of CAD, comparisons between MI and healthy controls means comparing CAD with non-CAD. Hence, it can be considered that *ANRIL* mainly influences coronary atherosclerosis, instead of MI/ACS, which is pathophysiologically distinct from CAD [37].

Risk factors for CAD are both environmental and genetic. PCAD has a stronger genetic susceptibility [38], and heritability of PCAD is more obvious than that of the LCAD [39]. We observed an appreciably higher OR for the risk alleles of *ANRIL* in PCAD individuals compared with the LCAD individuals. Previous evidence has reported the associations of 9p21 SNPs with PCAD in different populations [40, 41]. Our results may favor prediction of PCAD by providing new SNPs.

Epigenetics has been shown to play an important role in the initiation and progression of atherosclerosis. Atherosclerosis is even regarded as an epigenetic disease [42]. Among different epigenetic mechanisms, DNA methylation is the key epigenetic process for CAD and its risk factors [43]. Our results show that *p15*^*INK4b*^, but not *p14*^*ARF*^ or *p16*^*INK4a*^, is hypermethylated in CAD cases, consistent with the study of Zhuang et al*.* [19]. DNA methylation of *ANRIL* has been reported as an important epigenetic regulatory factor in mediating CAD [16], adiposity [18] and cardiovascular risk [17]. However, its relation with *ANRIL* polymorphism has not been reported. In the post-GWAS era, increasingly evidence has suggested a complex relationship between SNP, allele-specific binding of transcription factors, and allele-specific DNA methylation for disease risk [44].

Our results found that methylation levels of *ANRIL* are similar between the cases and controls (*p* > 0.05), but are different for the rs1004638 genotype, where AA and AT had a higher level of *ANRIL* methylation (*p* < 0.05). Furthermore, correlation analysis found *ANRIL* methylation levels are significantly associated with risk factors for CAD, and functional annotation indicates that the *ANRIL* methylated region has binding sites for transcription factors that are associated with basal transcription. These results showed there may be allele-related DNA methylation and allele-related binding of transcription factors within the *ANRIL* promoter. *ANRIL* may cause CAD via allele-related binding of transcription factors or allele-related gene expression. As for the role of allele-related DNA methylation, the mediator or the phenotype of CAD, further prospective studies are needed to explore this. Previous methylation quantitative-trait loci (met QTL) analyses also revealed that DNA methylation is under strong genetic influence [45] and most effective methylation is associated with nearby SNPs [46–48].

Methylation of *p15*^*INK4b*^ or *p14*^*ARF*^ is not affected by *ANRIL* genotype. Zhuang et al*.* also found that methylation of the *INK4/ARF* locus is not affected by SNPs in the *ANRIL* locus (typified by rs10757274) [19]. This led Lillycrop et al*.* to speculate that both risk genotype of *ANRIL* (rs10757274) and methylation of the *INK4/ARF* locus independently affect *ANRIL* expression to mediate disease risk [18]. We are in favor of this hypothesis.

### Strengths and limitations

The strengths of this study are the relatively large number of participants with definite diagnosis and detailed clinical characteristics. On the other hand, the genetic (genotype) and epigenetic (methylation) association analysis are from the same set of samples. There are some potential limitations. First, our study is only a preliminary study with just the results of association analysis. Further cellular and molecular experiments are warranted to validate the specific mechanism. Second, the epigenome is dynamic as the environment changes and due to the heterogeneity of patients and variations in therapies, so it is probably inappropriate to extend the present research results to other cells or tissues. Finally, our study is a case–control and cross-sectional design study. Whether methylation of *ANRIL* is a driver of CAD or a consequence of CAD requires further evaluation. Dynamic detection of *ANRIL* methylation before and after the occurrence of CAD would be a better choice.

## Conclusions

In conclusion, our findings indicate that polymorphisms of *ANRIL* (rs1004638, rs1333048 and rs1333050) might serve as a genetic biomarker of CAD, but not MI/ACS. There may be allele-related DNA methylation and allele-related binding of transcription factors within the *ANRIL* promoter. Our study provides new mechanistic insight into the regulation of *ANRIL*. Further studies are warranted to illustrate potential mechanisms for crosstalk of genetic factors, allele-related DNA methylation, and allele-related binding of transcription factors.

## Supplementary Information

**Additional file 1: Fig. S1.** Linkage disequilibrium structure and haplotype blocks of *ANRIL* in the Chinese population. **Table S1.** *ANRIL* tag SNP genotyping assay and Hardy–Weinberg equilibrium test. **Table S2.** Primer sequences for *ANRIL*, *P14*^*ARF*^, *P15*^*INK4b*^ and *p16*^*INK4a*^. **Table S3.** Genetic model analysis of the association of *ANRIL* tag SNPs with CAD risk. **Table S4.** Association of *ANRIL* tag SNPs with MI/ACS risk.

## Data Availability

All data supporting the findings of this study are available within the manuscript except for the raw sequence data. Any data providing genotype information is considered to be personal property by Chinese law, hence the submission to public achieves is prohibited. The raw sequence data can be acquired upon reasonable request from the authors (xubayi81@qq.com) if approval could be granted from the Ethics Committee of First Affiliated Hospital of Shantou University Medical College.

## Abbreviations

*ANRIL*: Antisense non-coding RNA in the INK4 locus
CAD: Coronary artery disease
PCAD: Premature CAD
LCAD: Late-onset CAD
MI: Myocardial infarction
ACS: Acute coronary syndrome
CVD: Cardiovascular disease
OR: Odds ratio
CI: Confidence interval
SD: Standard deviation
SNP: Single nucleotide polymorphism
SBP: Systolic blood pressure
DBP: Diastolic blood pressure
HbA1c: Hemoglobin A1c
TC: Total cholesterol
TG: Triglyceride
HDL: High-density lipoprotein cholesterol
LDL: Low-density lipoprotein cholesterol
TFBS: Transcription factor binding sites
