# Supplementary material for: Genetic and epigenetic associations of ANRIL with coronary artery disease and risk factors
Source: BMC Med Genomics. 2021 Oct 6;14:240. doi: 10.1186/s12920-021-01094-8 (PMC8496081; doi:10.1186/s12920-021-01094-8)
Supplement: Supplementary file 1 — Additional file 1: Fig. S1. Linkage disequilibrium structure and haplotype blocks of ANRIL in the Chinese population. Table S1. ANRIL tag SNP genotyping assay and Hardy–Weinberg equilibrium test. Table S2. Primer sequences for ANRIL, P14ARF, P15INK4b and p16INK4a. Table S3. Genetic model analysis of the association of ANRIL tag SNPs with CAD risk. Table S4. Association of ANRIL tag SNPs with MI/ACS risk. [file 12920_2021_1094_MOESM1_ESM.docx]

**Genetic and epigenetic associations of *ANRIL* with coronary artery disease and risk factors**

Bayi Xu^1^, Zhixia Xu^2^, Yequn Chen^1^, Nan Lu^1^, Zhouwu Shu^1^, Xuerui Tan^1 *^

^1^Department of Cardiology, First Affiliated Hospital of Shantou University Medical College, Shantou 515041, Guangdong, China.

^2^Medical Department, Second Affiliated Hospital of Shantou University Medical College, Shantou 515041, Guangdong, China.

*Correspondence to xueruitan@sina.com

^1^Department of Cardiology, First Affiliated Hospital of Shantou University Medical College, Shantou 515041, Guangdong, China


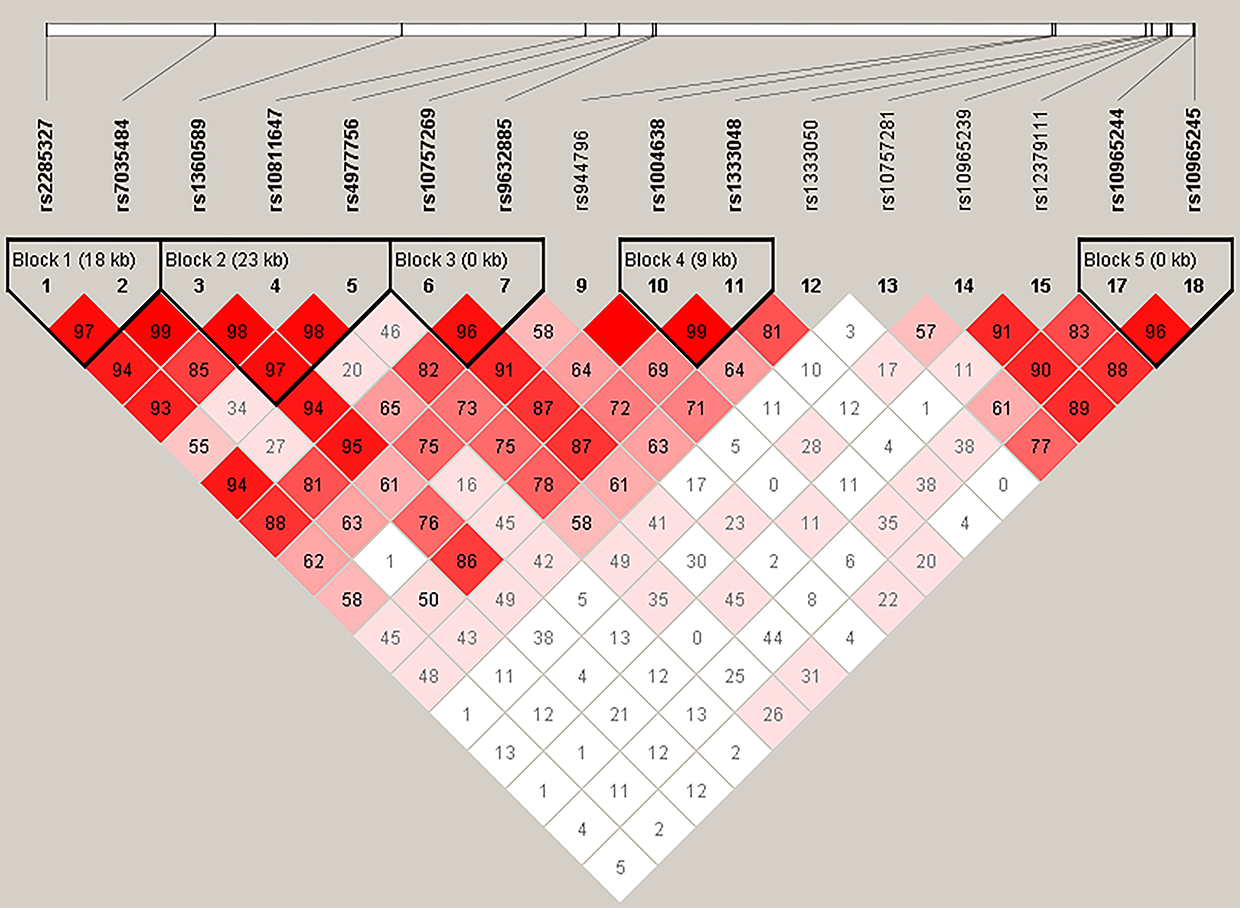


**Figure S1** Linkage disequilibrium structure and haplotype blocks of *ANRIL* in the Chinese population.

**Table S1** *ANRIL* tag SNP genotyping assay and Hardy-Weinberg equilibrium test.

| NO. | SNP | Genotyping success rate (%) | MAF | Alleles | H-W *p*-value | |
| --- | --- | --- | --- | --- | --- | --- |
|  |  |  |  |  | Case | Control |
| 1 | rs2285327 | 96.49 | 0.217 | A:G | 0.269 | 0.298 |
| 2 | rs7035484 | 99.33 | 0.340 | G:C | 0.849 | 0.956 |
| 3 | rs1360589 | 99.18 | 0.093 | A:G | 0.382 | 0.711 |
| 4 | rs10811647 | 99.55 | 0.479 | G:C | 0.011 | 0.450 |
| 5 | rs4977756 | 99.55 | 0.190 | A:G | 0.847 | 0.236 |
| 6 | rs10757269 | 95.82 | 0.189 | G:A | 0.612 | 0.465 |
| 7 | rs9632885 | 99.10 | 0.462 | A:G | 0.993 | 0.066 |
| 8 | rs10965227 | 99.25 | 0.147 | A:G | < 0.001 | < 0.001 |
| 9 | rs944796 | 99.33 | 0.220 | C:G | 0.748 | 0.208 |
| 10 | rs1004638 | 99.63 | 0.299 | A:T | 0.492 | 0.151 |
| 11 | rs1333048 | 99.25 | 0.497 | C:A | 0.909 | 0.371 |
| 12 | rs1333050 | 99.48 | 0.484 | T:C | 0.228 | 0.149 |
| 13 | rs10757281 | 99.70 | 0.183 | C:T | 0.440 | 0.907 |
| 14 | rs10965239 | 99.33 | 0.231 | A:G | 0.920 | 0.355 |
| 15 | rs12379111 | 98.88 | 0.150 | C:G | 0.918 | 1.000 |
| 16 | rs10965241 | 99.10 | 0.147 | G:C | < 0.001 | < 0.001 |
| 17 | rs10965244 | 99.70 | 0.119 | A:T | 0.766 | 1.000 |
| 18 | rs10965245 | 99.40 | 0.401 | G:A | 0.394 | 0.126 |

MAF, Minor allele frequency; H-W, Hardy-Weinberg equilibrium test.

**Table S2** Primer sequences for *ANRIL*, *P14^ARF^*, *P15^INK4b^* and *p16^INK4a^.*

| Gene | Primer | sequence |
| --- | --- | --- |
| *ANRIL* | ANRIL-F | AATATGGTGAGTAGGTTTTTGGTGATTT |
|  | ANRIL-R | ACCCTAAACCCTAACTCCTCAATAACATC |
|  | ANRIL-S | AAACCACCAAAACCCTC |
| *P14^ARF^* | P14-F | GGGGGAGGATTTTTTTTTAATAGAGTG |
|  | P14-R | CCCCTAAAATCCCCCCAAATTAAATCTC |
|  | P14-S | CACCTACCCCCCACA |
| *P15^INK4b^* | P15-F | GTTTGGATTGTTTTTGGGAAAAAG |
|  | P15-R | ACTCCCCACTCTACCAAA |
|  | P15-S | GGAAGTAGTAGAGTTTAAAGT’ |
| *p16^INK4a^* | P16-F | TGGAGGTTAGGGTGGGAGT |
|  | P16-R | CACCCCCCCCTACCCATCT |
|  | P16-S | TGGGGGTGGGGGTGA |

F: forward primer; R: reverse primer; S: sequencing primer

**Table S3** Genetic model analysis of the association of *ANRIL* tag SNPs with CAD risk.

| SNP | Model | | Case, n(%) | Control, n(%) | χ^2^ | *P-*value |
| --- | --- | --- | --- | --- | --- | --- |
| rs1004638 | Genotype | TT | 27(5.39) | 68(13.57) | 21.46 | <0.001 |
|  |  | AT | 206(41.12) | 208(41.52) |  |  |
|  |  | AA | 268(53.49) | 225(44.91) |  |  |
|  | Recessive | TT | 27(5.39) | 68(13.57) | 19.55 | <0.001 |
|  |  | AT+AA | 474(94.61) | 433(86.43) |  |  |
|  | Dominant | TT+AT | 233(46.51) | 276(55.09) | 7.38 | 0.007 |
|  |  | AA | 268(53.49) | 225(44.91) |  |  |
| rs1333048 | Genotype | AA | 104(20.80) | 141(28.37) | 16.02 | <0.001 |
|  |  | AC | 249(49.80) | 259(52.11) |  |  |
|  |  | CC | 147(29.40) | 97(19.52) |  |  |
|  | Recessive | AA | 104(20.80) | 141(28.37) | 7.71 | 0.006 |
|  |  | AC+CC | 396(79.20) | 356(71.63) |  |  |
|  | Dominant | AA+AC | 353(70.60) | 400(80.48) | 13.17 | <0.001 |
|  |  | CC | 147(29.40) | 97(19.52) |  |  |
| rs1333050 | Genotype | CC | 101(20.12) | 124(24.90) | 6.74 | 0.034 |
|  |  | CT | 259(51.59) | 265(53.21) |  |  |
|  |  | TT | 142(28.29) | 109(21.89) |  |  |
|  | Recessive | CC | 101(20.12) | 124(24.90) | 3.28 | 0.070 |
|  |  | CT+TT | 401(79.88) | 374(75.10) |  |  |
|  | Dominant | CC+CT | 360(71.71) | 389(78.11) | 5.45 | 0.002 |
|  |  | TT | 142(28.29) | 109(21.89) |  |  |
| rs4977756 | Genotype | AA | 335(66.73) | 313(62.73) | 6.86 | 0.032 |
|  |  | AG | 154(30.68) | 157(31.46) |  |  |
|  |  | GG | 13(2.59) | 29(5.81) |  |  |
|  | Recessive | AA | 335(66.73) | 313(62.73) | 1.76 | 0.185 |
|  |  | AG+GG | 167(33.27) | 186(37.27) |  |  |
|  | Dominant | AA+AG | 489(97.41) | 470(94.19) | 6.46 | 0.011 |
|  |  | GG | 13(2.59) | 29(5.81) |  |  |
| rs9632885 | Genotype | AA | 158(31.73) | 120(24.10) | 7.64 | 0.022 |
|  |  | AG | 237(47.59) | 272(54.62) |  |  |
|  |  | GG | 103(20.68) | 106(21.29) |  |  |
|  | Recessive | AA | 158(31.73) | 120(24.10) | 7.21 | 0.007 |
|  |  | AG+GG | 340(68.27) | 378(75.90) |  |  |
|  | Dominant | AA+AG | 395(79.32) | 392(78.71) | 0.05 | 0.815 |
|  |  | GG | 103(20.68) | 106(21.29) |  |  |

**Table S4** Association of *ANRIL* tag SNPs with MI/ACS risk.

| **SNP** | **Genotype** | **MI/ACS**  **(n=188)** | **CAD without**  **MI/ACS**  **(n=188)** | ***P-*value** |
| --- | --- | --- | --- | --- |
| rs1004638 | AA | 101(53.72) | 100(53.19) | 0.673 |
|  | AT | 78(41.49) | 75(39.89) |  |
|  | TT | 9(4.79) | 13(6.91) |  |
| rs1333048 | AA | 35(18.62) | 46(24.47) | 0.343 |
|  | AC | 99(52.66) | 88(46.81) |  |
|  | CC | 54(28.72) | 54(28.72) |  |
| rs1333050 | CC | 34(18.09) | 44(23.40) | 0.440 |
|  | CT | 97(51.60) | 92(48.94) |  |
|  | TT | 57(30.32) | 52(27.66) |  |

Values are n (%).
